# Predictive value of HBeAg titer dynamics for HBsAg clearance in pediatric chronic hepatitis B

**DOI:** 10.3389/fped.2025.1539300

**Published:** 2025-04-03

**Authors:** Sukjin Hong, Jun Hyun Hwang, Keumoung Kim, Younghae Do, Naeun Kwak, Hyo Rim Suh, Sujin Choi, Ben Kang, Byung-Ho Choe

**Affiliations:** ^1^ Department of Pediatrics, School of Medicine, Daegu Catholic University, Daegu, Republic of Korea; ^2^ Department of Preventive Medicine, School of Medicine, Daegu Catholic University, Daegu, Republic of Korea; ^3^ Department of Mathematics, Nonlinear Dynamics & Mathematical Application Center, Kyungpook National University, Daegu, Republic of Korea; ^4^ Department of Pediatrics, School of Medicine, Kyungpook National University, Daegu, Republic of Korea

**Keywords:** hepatitis B surface antigens, hepatitis B e antigens, seroconversion, antiviral agents, children

## Abstract

**Introduction:**

Achieving functional cure of chronic hepatitis B (CHB), characterized by the loss of HBV DNA and HBsAg, remains challenging in adults but demonstrates higher success rates in children. Elucidating the factors influencing HBsAg loss in pediatric patients is crucial for optimizing treatment strategies. This study aimed to evaluate the predictive value of HBeAg titer dynamics for HBsAg clearance in pediatric CHB and develop a predictive model incorporating these dynamics.

**Material and methods:**

This retrospective cohort study analyzed 119 children aged 1–18 years with CHB treated with nucleos(t)ide analogues. Patient outcomes were evaluated using two independent classification approaches: HBsAg loss status and age stratification (≤6 vs. >6 years). Treatment response was assessed through longitudinal HBeAg titer measurements during the first 12 months. Based on identified predictors, a logistic regression model was developed incorporating age and HBeAg titer dynamics to predict HBsAg clearance probability.

**Results:**

The study population exhibited a median age of 6.2 years. Factors associated with HBsAg loss encompassed younger age, female sex, and absence of breakthrough. In multivariate analysis, younger age was identified as the only significant factor. The cumulative HBsAg loss rate demonstrated markedly higher values in the ≤6 years group (Hazard ratio 7.69). HBeAg titer decline exhibited significantly steeper trajectories in the HBsAg loss group. The developed predictive model, “Log Odds = −1.182 + 0.308 × log_reduction−0.205 × age”, demonstrated good performance with high accuracy.

**Conclusions:**

Early HBeAg titer dynamics combined with age at treatment initiation may serve as useful predictors of HBsAg clearance in pediatric CHB. Our predictive model, utilizing readily available semi-quantitative HBeAg measurements, could potentially assist clinicians in therapeutic decision-making and individualized treatment strategies.

## 1 Introduction

The ultimate therapeutic goal in hepatitis B treatment encompasses the complete eradication of HBV DNA and HBsAg from the blood, coupled with the elimination of covalently closed circular DNA (cccDNA) from liver tissue. However, achieving this complete cure presents significant challenges in current clinical practice. Therefore, the current therapeutic focus centers on achieving a “functional cure,” defined as the loss of HBV DNA and HBsAg in the blood. Although cccDNA may persist, functional cure demonstrates significant clinical benefits, including no relapse post-treatment, no progression of liver damage, and reduced risk of cirrhosis and hepatocellular carcinoma (1).

In contemporary clinical practice, maintaining undetectable serum HBV DNA levels and normal liver function tests are considered indicative of good treatment responses, as functional cure remains elusive even with prolonged nucleos(t)ide analogue (NUCs) treatment (2, 3). While the functional cure rate in adults remains below 5%, pediatric CHB patients demonstrate notably higher success rates. Recent data from Asian populations indicate functional cure rates ranging from 40% to 50% in children under seven years of age (4, 5).

Previous studies, including our earlier work (Choe at al.), have demonstrated that children under the age of six exhibit higher clearance rates of HBsAg compared to older children with HBeAg-positive CHB (4, 6, 7). While the age-dependent nature of HBsAg clearance is well documented, HBeAg titer dynamics during treatment could potentially serve as a predictor, but their prognostic value remains unexplored. Therefore, this study aimed to investigate whether early changes in HBeAg titer, combined with age-related factors, could provide a reliable framework for predicting HBsAg clearance, thereby enabling early identification of patients most likely to achieve functional cure.

## 2 Materials and methods

### 2.1 Study design and population

**Figure 1:**
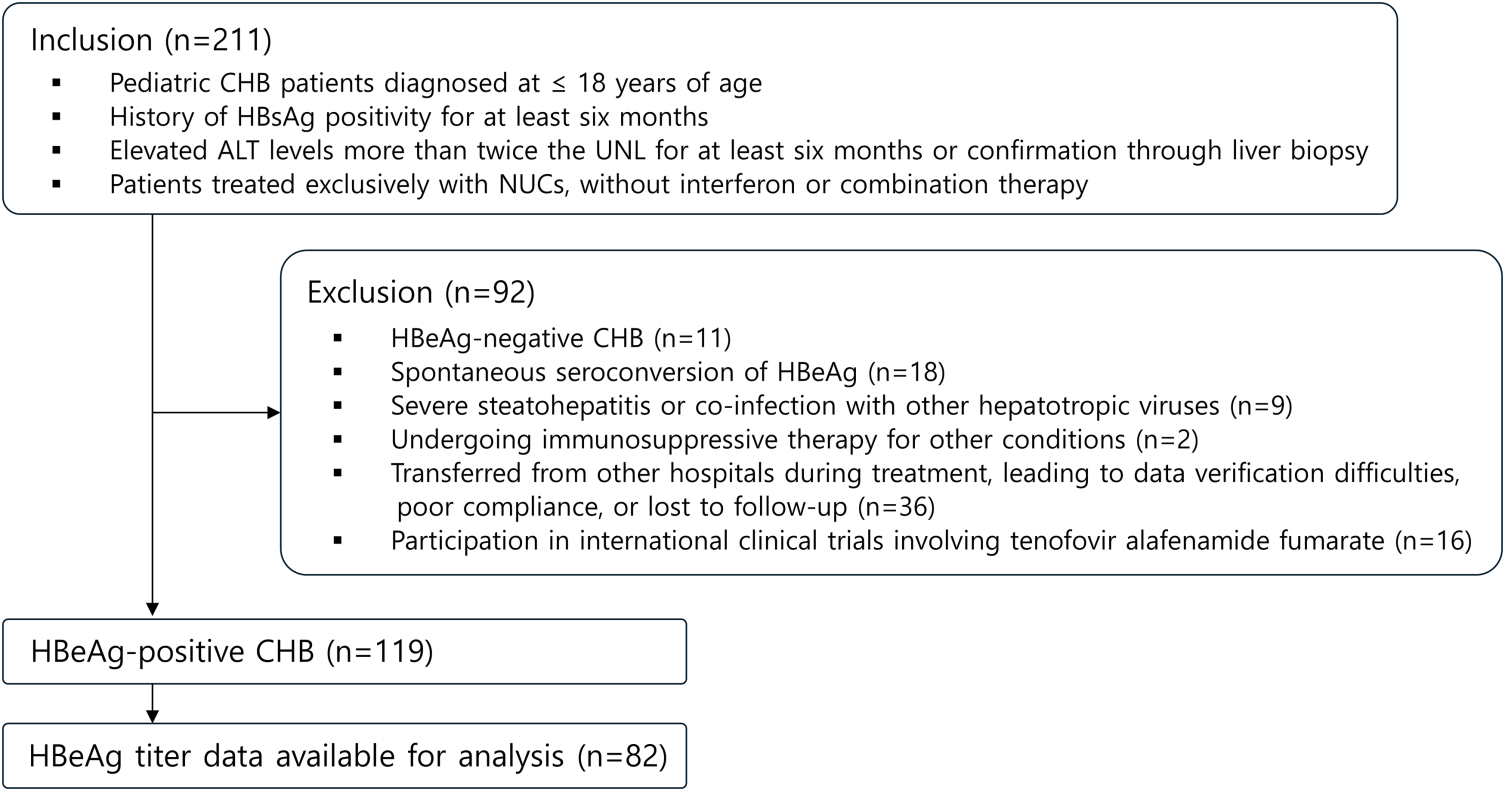
Flowchart outlining study design. CHB, chronic hepatitis B; UNL, upper normal limit; NUCs, nucleos(t)ide analogues.

This retrospective cohort investigation analyzed data from pediatric CHB patients who presented to our institution between March 30, 1999, and December 30, 2022. The study population comprised 211 children aged 1–18 years with CHB. Following the application of exclusion criteria, 119 patients were included in the final analysis (Figure 1). The follow-up duration ranged from 3 to 5 years post-treatment initiation, with exceptions made for patients who achieved HBsAg loss earlier. For cases of treatment discontinuation without achieving complete remission (CR)—defined as undetectable HBV DNA levels, HBeAg seroconversion, and normalization of ALT levels—or HBsAg loss, only patients with a minimum one-year post-treatment observation period were included.

### 2.2 Treatment protocols

Treatment protocols employed three primary nucleos(t)ide analogues (NUCs) as first-line therapy: lamivudine, entecavir, and tenofovir. Second-line therapeutic options, implemented in cases requiring medication changes, comprised adefovir, entecavir, and tenofovir, administered either as monotherapy or in combination. Antiviral regimens were selected based on age-specific approvals by the Korean Food and Drug Administration (KFDA): lamivudine for patients of all ages throughout the study period, adefovir for patients aged ≥12 years since 2005, entecavir for patients aged ≥16 years since 2007 and ≥2 years since 2016, and tenofovir for patients aged ≥12 years since 2012. Treatment continuation protocols extended beyond CR, incorporating consolidation therapy for a minimum of 1 year. Recent guideline revisions by The Asian Pacific Association for the Study of the Liver (APASL) and The European Association for the Study of the Liver (EASL) have extended the recommended consolidation therapy duration to over 3 years (2, 8, 9).

### 2.3 Serological assays

Serological testing methods evolved throughout the study period. For HBeAg, HBsAg, anti-HBe, and anti-HBs detection, both electrochemiluminescence immunoassay (ECLIA method, Roche Diagnostics Corporation, Indianapolis, IN) and enzyme immunoassay (Behringer ELISA Processor III, Dade Behringer Diagnostics, Marburg, Germany by 1999) were employed. HBeAg quantification utilized sample to cut-off value ratios (S/CO), with positivity defined as S/CO ≥ 1.0, using electrochemiluminescence immunoassay (ECLIA) on cobas e immunoassay analyzers.

HBV DNA quantification methods progressed through three generations of testing platforms. Initial measurements employed the hybrid capture II HBV DNA assay (DML 2000 system, Digene, Gaithersburg, MD) through 2004, with a detection limit of 0.5 pg/ml (≈25,000 IU/ml). From January 2005, the branched DNA signal amplification assay (VERSANT 3.0, Bayer HealthCare LLC, Tarrytown, NY, USA) was implemented, offering enhanced sensitivity with a detection limit of 0.007 pg/ml (≈357 IU/ml). Further improvement in detection capability was achieved from May 2008 with the introduction of real-time PCR (Roche COBAS AmpliPrep/COBAS TaqMan96 real-time PCR assay, Roche Diagnostics, Mannheim, Germany), providing a detection limit of 12 IU/ml.

HBV genotyping was performed by nested PCR amplification of the polymerase reverse transcriptase region using primers POL-RT-F1/POL-RT-R1 and POL-RT-F2/POL-RT-R2, yielding a 1,291-bp fragment. PCR products were purified with the MEGA quick-spin Fragment DNA purification kit (Intron Biotechnology, Seoul, Korea). Purified products were cloned into the TOPO TA cloning vector (Invitrogen, Carlsbad, CA, USA), and sequencing was conducted on an ABI 373A DNA sequencer (Applied Biosystems, Foster City, CA, USA). HBV genotypes were assigned by phylogenetic analysis of the 1,032-bp RT region and recombination breakpoints were determined by BootScan analysis using the recombination detection program.

### 2.4 Analysis of HBsAg loss predictors

The investigation encompassed comprehensive analyses of patient characteristics in relation to clinical outcomes, with particular emphasis on HBsAg loss. In cases of HBsAg clearance, confirmation was obtained through quantitative HBsAg testing. The study population was analyzed using two independent classification approaches: HBsAg loss status (loss vs. non-loss) and age stratification (≤6 years vs. >6 years). The analytical framework incorporated both categorical and continuous variables. Categorical variables encompassed sex, initial antiviral therapy, and breakthrough status during treatment. The operational definition of “breakthrough” incorporated both virologic and biochemical breakthrough events. A virologic breakthrough is the re-emergence or surge of HBV DNA during therapy (commonly defined as an increase of more than 1 − log10 IU/ml from the lowest value during therapy), and a biochemical breakthrough is the return of elevated ALT levels after they had normalized on therapy (8). Continuous variables included age, ALT, HBV DNA, and HBeAg titer at treatment initiation. HBV DNA values underwent logarithmic transformation (log^10^) prior to analysis.

### 2.5 Analysis using HBeAg titer

The analytical strategy focused on evaluating treatment response through semi-quantitative HBeAg titer measurements. Patient monitoring intervals ranged from monthly to several-month periods, with HBeAg titer assessment at each visit. To establish early response patterns, analysis was restricted to the initial 12-month post-treatment period. HBeAg titer data underwent log transformation to achieve normal distribution. Quantitative assessment of treatment response utilized average slope calculations of HBeAg titer reduction. To ensure comparative accuracy, initial HBeAg titer values served as covariates for adjusting individual patient log reduction rates. Visual representation of HBeAg titer dynamics employed log scale graphs for each group.

### 2.6 Development of predictive model

Based on the findings from initial analyses, a predictive model was developed incorporating age at treatment initiation and adjusted log reduction rates as independent variables. The model's performance was assessed through comprehensive statistical evaluation.

### 2.7 Statistical analysis

Statistical analysis incorporated several methodological approaches. Categorical variables were summarized using frequency distributions and analyzed via chi-square test, while continuous variables were characterized by median and interquartile range (IQR) and compared using Mann–Whitney *U* test. HBsAg loss risk assessment employed Cox proportional hazards regression models with multivariate analysis for potential confounders. Survival analysis of cumulative HBsAg loss rates between age groups utilized the Kaplan–Meier estimator for survival function visualization, with log-rank test for survival distribution comparison. The Cox Proportional Hazards model assessed age group impact on outcomes. For HBeAg titer reduction analysis, simple linear regression calculated average slopes, with independent samples *t*-test for group comparisons. The predictive model was developed using logistic regression through the glm function in R. Model performance was assessed through ROC curve analysis, with AUC calculation and optimal threshold determination via Youden's J statistic. Statistical significance was established at *p* < 0.05. All analyses were conducted using R statistical software, version 4.4.1 (R Foundation for Statistical Computing).

## 3 Results

### 3.1 Demographics and clinical data of the study cohort

Baseline characteristics of the study population are summarized in Table 1. The study cohort demonstrated a median age of 6.20 years, with male participants comprising 55.5% (66/119) of the population. Among patients achieving therapeutic milestones, the median time to complete remission (CR) was 12 months (*n* = 93), while HBsAg loss occurred at a median of 17 months (*n* = 33). Treatment protocols predominantly utilized lamivudine as initial therapy (80.7%, 96/119). Treatment breakthrough events occurred in 28 patients (23.5%), exclusively among those receiving lamivudine. HBV genotyping was performed only in patients experiencing breakthrough events (*n* = 28), and all were identified as genotype C (Table 1). Vertical transmission, confirmed by maternal HBV carrier status, accounted for 89% (106/119) of cases. Analysis of age distribution between transmission routes revealed comparable median IQRs: maternal HBsAg-positive group: 6.05 (1.3–11.725), maternal HBsAg-negative group: 6.8 (5.5–11.05) (*p* = 0.494).

**Table 1:** Baseline characteristics at treatment initiation (*n* = 119).

Characteristic	Category	Value
Age, years		6.20 (2.35–12.65)
ALT, U/L		137 (105–225.5)
HBV DNA, log_10_IU/ml		7.30 (7.11–7.74)
HBeAg titer, S/CO (*n* = 82)		1,108 (607–1,429)
Elapsed time to CR, months (*n* = 93)		12 (8–20)
Elapsed time to HBsAg loss, months (*n* = 33)		17 (10–29)
Sex	Male	66 (55.5%)
	Female	53 (44.5%)
Initial Antiviral Therapy	Lamivudine	96 (80.7%)
	Entecavir	12 (10.1%)
	Tenofovir	11 (9.2%)
Breakthrough (*n* = 28)	Yes	28 (23.5%)
	No	91 (76.5%)
HBV genotype (among breakthrough patients, *n* = 28)		Genotype C: 28 (100%)

Categorical variables expressed as number (n) and percentage (%), and continuous variables as median (interquartile range). HBV, hepatitis B virus; ALT, alanine aminotransferase; HBsAg, hepatitis B surface antigen; HBeAg, hepatitis B e antigen.

### 3.2 Characteristics associated with HBsAg loss

Baseline characteristics and clinical outcomes stratified by HBsAg loss status are summarized in Table 2. Comparative analysis of baseline characteristics revealed markedly lower age at treatment initiation in the HBsAg loss group (median: 2.3 years vs. 9.7 years). Initial ALT levels were higher in the HBsAg loss group (median: 156 U/L vs. 127 U/L; *p* = 0.227), though not reaching statistical significance. Baseline HBV DNA levels and HBeAg titers showed no significant differences between groups. Gender distribution analysis revealed a significantly higher proportion of females in the HBsAg loss group (63.6% vs. 37.2%). While initial antiviral therapy protocols showed comparable distribution between groups, breakthrough events demonstrated significant disparity, occurring in only 3.0% of the HBsAg loss group compared to 31.4% in the non-HBsAg loss group (Table 2).

**Table 2:** Comparison of HBsAg loss group and Non-HBsAg loss group at treatment initiation.

Variable	HBsAg loss (*N* = 33)	Non-HBsAg loss (*N* = 86)	Univariate analysis (*p*-value)
Age, years	2.3 (1.3–4.3)	9.7 (4.725–13.25)	**<0**.**001**
ALT, U/L	156 (115–238)	127 (105–203.5)	0.227
HBV DNA, log_10_IU/ml	7.30 (7.18–7.73)	7.30 (7.09–7.74)	0.774
HBeAg titer, S/CO	1,173 (779–1,479.5)	1,091.5 (489.75–1,386.75)	0.302
Sex			**0**.**017**
Male	12 (36.4%)	54 (62.8%)	
Female	21 (63.6%)	32 (37.2%)	
Initial Antiviral Therapy			0.334
Lamivudine	28 (84.8%)	68 (79.1%)	
Entecavir	4 (12.1%)	8 (9.3%)	
Tenofovir	1 (3.0%)	10 (11.6%)	
Breakthrough			**0**.**002**
Yes	1 (3.0%)	27 (31.4%)	
No	32 (97.0%)	59 (68.6%)	

Categorical variables expressed as number (n) and percentage (%), and continuous variables as median (interquartile range). Statistically significant *p*-values are indicated in bold. HBV, hepatitis B virus; ALT, alanine aminotransferase; HBsAg, hepatitis B surface antigen; HBeAg, hepatitis B e antigen.

Multivariate Cox regression analysis identified age at treatment initiation as the sole independent significant factor (HR: 0.79, 95% CI 0.70–0.88). Female gender demonstrated a positive association with HBsAg loss, though not reaching statistical significance (HR: 1.77, 95% CI: 0.87–3.62, *p* = 0.15). The absence of breakthrough events suggested a favorable impact on HBsAg loss, approaching statistical significance (HR: 7.0, 95% CI: 0.95–51.70, *p* = 0.054) (Table 3). Among patients achieving HBsAg loss, 91% (30/33) subsequently developed anti-HBs antibodies after a median interval of 7.0 months (IQR: 0.5–14.0) following HBsAg loss, while anti-HBs antibodies were detected simultaneously with the loss of HBsAg in seven patients.

**Table 3:** Multivariate Cox analysis of factors affecting HBsAg loss.

Variable	HR	95% CI	*p*-value
Age (years)	0.79	0.70–0.88	**<0**.**001**
ALT (U/L)	1.0	0.99–1.0	0.589
HBV DNA (log_10_IU/ml)	0.71	0.40–1.28	0.258
Sex (Ref: male)	1.77	0.87–3.62	0.150
Initial antiviral therapy (Ref: Lamivudine)			
Entecavir	0.83	0.39–1.79	0.637
Tenofovir	0.53	0.06–4.29	0.555
Breakthrough (Ref: Yes)	7.0	0.95–51.70	0.054

Statistically significant *p*-values are indicated in bold. HBV, hepatitis B virus; ALT, alanine aminotransferase; Ref, reference.

### 3.3 Age-related treatment outcomes

Stratification by age revealed distinct therapeutic responses between groups (Table 4). The younger cohort (≤6 years) demonstrated a median treatment initiation age of 2.3 years, compared to 12.6 years in the older group. Time to therapeutic milestone achievement showed significant age-dependent variation, with the younger group reaching CR more rapidly (median: 10 vs. 14 months) and achieving HBsAg loss earlier (median: 15.5 vs. 33 months).

**Table 4:** Comparison of characteristics at treatment initiation by age group.

Variable	Age ≤6 (*N* = 58)	Age >6, ≤ 18 (*N* = 61)	Univariate analysis (*p*-value)
Age, years	2.3 (1.3–4.3)	12.6 (9.8–13.9)	
Elapsed time to CR, months	10 (6–14)	14 (10–27)	**<0**.**001**
Elapsed time to HBsAg loss, months	15.5 (9.8–21.8)	33 (18–46)	**<0**.**001**
ALT, U/L	151.5 (116–238.8)	125 (103–199)	0.086
HBV DNA, log_10_IU/ml	7.3 (7.1–7.6)	7.3 (7.2–7.8)	0.627
HBeAg titer, S/CO	1,144 (770–1,480.25)	1,075 (419.5–1,266)	0.723
Sex			0.085
Male	27 (46.55%)	39 (63.93%)	
Female	31 (53.45%)	22 (36.07%)	
Initial Antiviral Therapy			**0**.**02**
Lamivudine	50 (86.21%)	46 (75.41%)	
Entecavir	7 (12.07%)	5 (8.20%)	
Tenofovir	1 (1.72%)	10 (16.39%)	
Breakthrough			**0**.**008**
Yes	7 (12.07%)	21 (34.43%)	
No	51 (87.93%)	40 (65.57%)	

Categorical variables expressed as number (n) and percentage (%), and continuous variables as median (interquartile range). Statistically significant *p*-values are indicated in bold. CR, complete remission; HBV, hepatitis B virus; ALT, alanine aminotransferase; HBsAg, hepatitis B surface antigen; HBeAg, hepatitis B e antigen.

**Figure 2:**
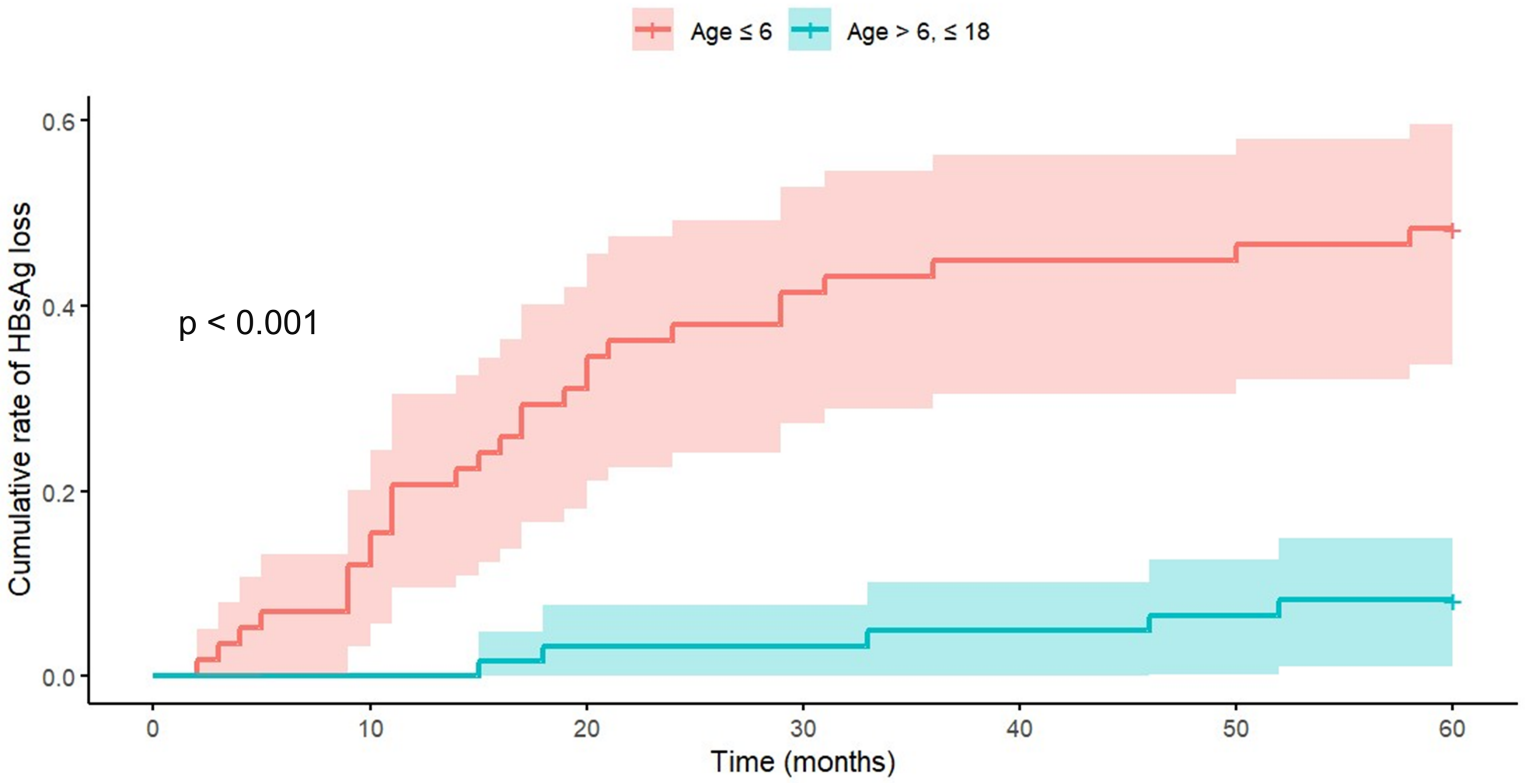
Age-dependent analysis of cumulative HBsAg loss rates. Kaplan–Meier survival analysis demonstrates significantly enhanced clearance rates in the younger cohort. Cox Proportional Hazards Model analysis revealed a hazard ratio of 7.69 (95% CI: 3.03–20.0, *p* < 0.001) for the Age ≤6 group. The visualization employs Kaplan–Meier curves to illustrate age-group differences.

Notably, baseline laboratory parameters (ALT levels, HBV DNA levels, and HBeAg titers) showed no significant age-related differences. Gender distribution analysis suggested a higher proportion of females in the younger age group (53.45% vs. 36.07%; *p* = 0.085). Initial therapeutic protocols demonstrated age-related variation, with higher tenofovir utilization in the older age group. Breakthrough events occurred significantly more frequently in the older age group (34.43% vs. 12.07%). All 28 breakthrough events occurred exclusively in lamivudine-treated patients, with 6 patients aged under 6 years (median: 3.3 years) and 22 patients aged ≥6 years (median: 12.3 years). Kaplan–Meier survival analysis demonstrated superior HBsAg clearance rates in the younger cohort (Figure 2). Cox proportional hazards modeling quantified this age-dependent effect, with the younger group demonstrating significantly higher clearance probability (HR: 7.69, 95% CI: 3.03–20.0, *p* < 0.001).

### 3.4 HBeAg titer dynamics and treatment response

**Figure 3:**
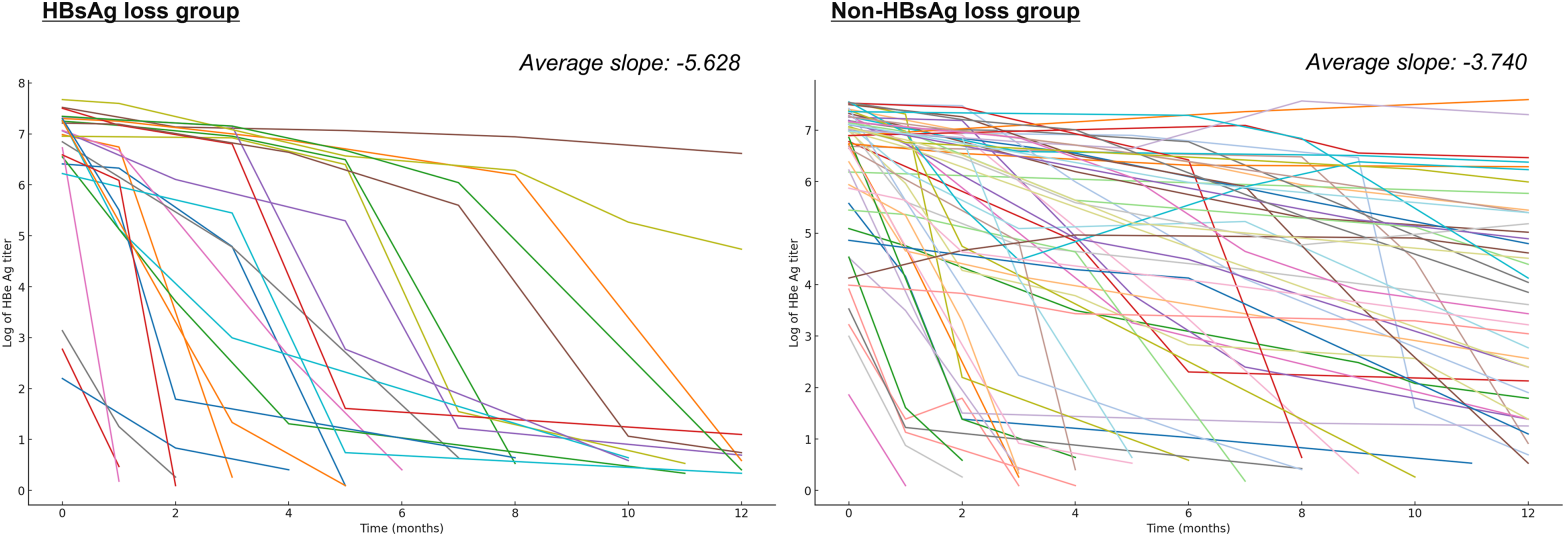
Comparative analysis of HBeAg titer reduction patterns. The illustration presents logarithmic HBeAg titer reduction trajectories for both outcome groups. The HBsAg loss group demonstrated an average slope of −5.628, in contrast to −3.740 in the non-HBsAg loss group (*t*-statistic = −3.982, *p* < 0.001).

Analysis of HBeAg titer kinetics revealed distinct patterns between outcome groups. The HBsAg loss group demonstrated significantly steeper decline rates (average slope: −5.628 vs. −3.740, *t*-statistic = −3.982, *p* < 0.001) (Figure 3). Early HBeAg negativization rates (within 12 months) showed marked differences: 87.0% in the HBsAg loss group vs. 37.3% in the non-HBsAg loss group (Table 5). Notably, patients failing to achieve CR uniformly demonstrated persistent HBeAg positivity at 12 months.

**Table 5:** Group comparison of 12-month HBeAg negativization.

Group	HBeAg loss within 12 months	No HBeAg loss within 12 months	*p*-value
HBsAg loss group (*N* = 23)	20 (47.6%)	3 (7.5%)	
Non-HBsAg loss group (*N* = 59)	22 (52.4%)	37 (92.5%)	
Total (*N* = 82)	42 (100%)	40 (100%)	*P* < 0.001

HBeAg, hepatitis B e antigen; HBsAg, hepatitis B surface antigen.

### 3.5 Predictive model performance

**Figure 4:**
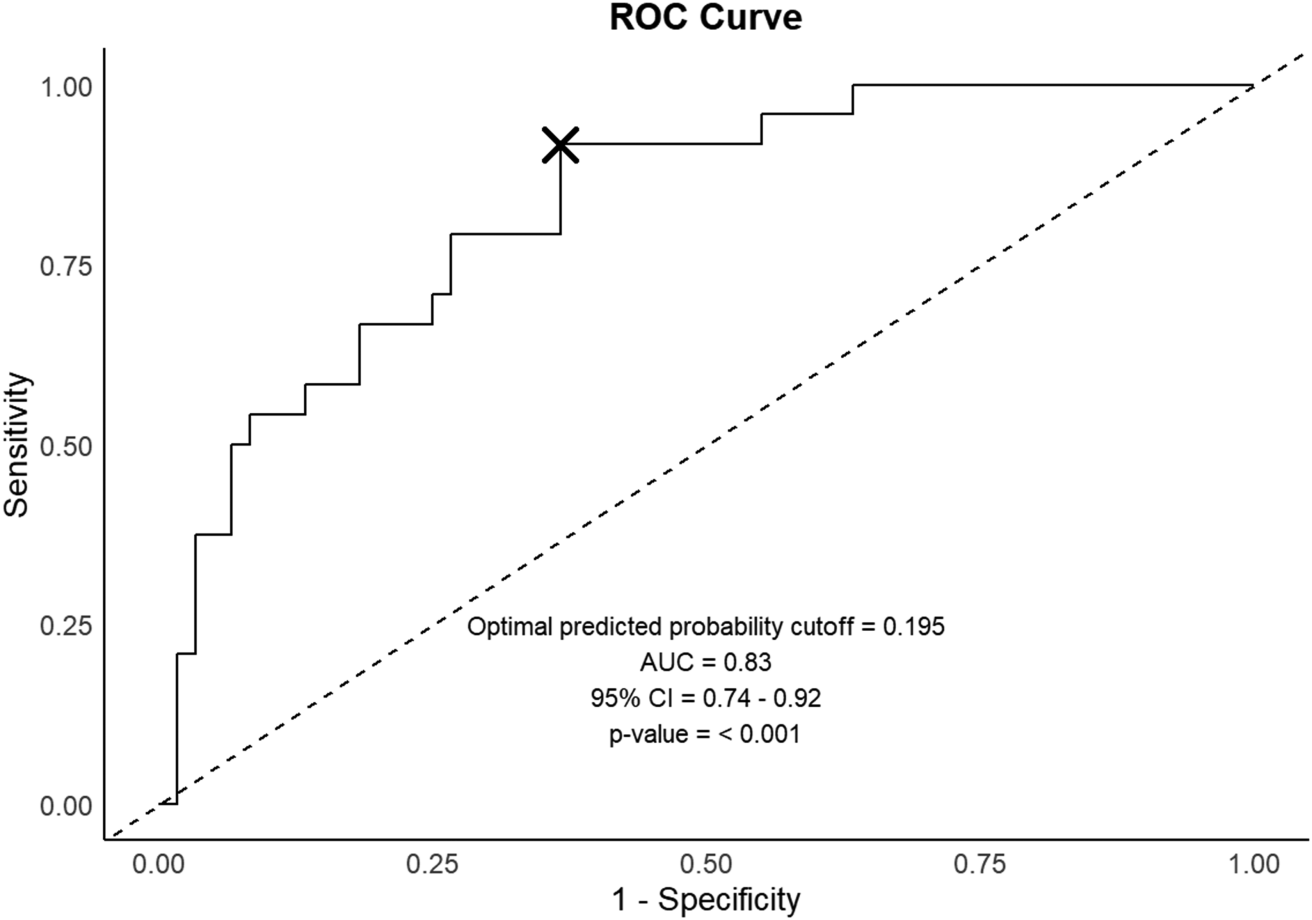
Predictive model performance evaluation. The ROC curve illustrates the model's discriminative capability, with optimal prediction threshold denoted by an x-mark. ROC, Receiver Operating Characteristic; AUC, area under the curve; CI, confidence interval.

The developed logistic regression model incorporating age and HBeAg titer dynamics is presented in Table 6. The model demonstrated robust predictive capability with an AUC of 0.83 (95% CI: 0.78–0.88, *p* < 0.001). Using the optimal prediction threshold of 0.195, the model achieved a sensitivity of 91.7% and specificity of 63.3% for predicting HBsAg loss. These performance metrics suggest that the model could serve as a valuable clinical tool for identifying patients with high probability of achieving HBsAg clearance (Figure 4).

**Table 6:** Logistic regression model for predicting HBsAg loss.

Log odds	−1.182 + 0.308 × log_reduction − 0.205 × age
Where:	
log_reduction	ln(1st titer + 1) − ln(2nd titer + 1)
age	Age at treatment initiation
1st titer	Initial HBeAg titer value at the start of treatment
2nd titer	HBeAg titer value at 12 months or at the time of negativization within 12 months
Probability	1/(1 + *e*^−Log Odds^)

HBeAg, hepatitis B e antigen; HBsAg, hepatitis B surface antigen.

## 4 Discussion

This study introduced a novel approach to predicting HBsAg clearance through systematic analysis of HBeAg titer dynamics in pediatric CHB patients. HBeAg titer patterns emerged as powerful predictors of therapeutic response. While previous studies have primarily relied on qualitative HBeAg status, our findings suggest that semi-quantitative HBeAg dynamics may have prognostic value. Early HBeAg negativization, especially within the first treatment year, was associated with favorable therapeutic response. The clinical utility of quantitative HBeAg measurement is supported by multiple lines of evidence. In HBeAg-positive patients receiving NUC therapy, lower on-treatment HBeAg levels and greater declines in HBeAg have been demonstrated to be highly predictive of treatment response (10, 11). Additional studies have demonstrated that semi-quantitative HBeAg measurement effectively reflects relative HBV DNA levels (12) and strongly correlates with quantitative HBeAg levels (13). Our findings extend this understanding by establishing HBeAg dynamics as a valuable tool for early treatment response prediction.

Based on these HBeAg dynamics insights, we developed a predictive model incorporating both HBeAg titer log reduction and patient age at treatment initiation. This approach may help clinicians estimate the likelihood of HBsAg clearance. For practical implementation, patient case examples are provided in the Supplementary Materials, complemented by an online calculator, which clinicians may find useful as a supportive tool in treatment decision-making.

Recent studies have consistently highlighted age at treatment initiation as a critical predictor of treatment outcomes in pediatric CHB, demonstrating superior therapeutic responses in younger children (Wang et al., Wu et al., Zhang et al.) (5, 14, 15). Although our findings are consistent with these studies in emphasizing the importance of younger age, notable methodological differences exist. While previous studies primarily evaluated outcomes following interferon-alpha, NUCs monotherapy, or combination therapy, our study focused exclusively on NUC monotherapy. Additionally, prior studies mainly relied on conventional clinical predictors, such as baseline ALT and HBV DNA levels, whereas our study utilized the dynamics of HBeAg titers as a novel predictor of treatment outcomes.

Our findings in a relatively young patient cohort (median age 6.2 years, IQR: 2.35–12.65) align well with data from Zhang et al. (median age 4.6 years, IQR: 2.68–8.22) (5) and Wu et al., showing a clear linear relationship between younger age at treatment initiation and higher rates of HBsAg loss and HBeAg clearance (14). Another recent study reported substantial functional cure rates (56.25%) even among young children in the immune-tolerant phase, further supporting the favorable therapeutic outcomes observed in younger pediatric patients (16). The superior treatment responses observed in younger pediatric patients may be explained by multiple factors. Immunologically, younger children exhibit higher absolute numbers and proportions of HBV-specific T and B lymphocytes, enabling stronger antiviral responses (6, 17). Additionally, younger children typically have lower viral burdens, fewer HBV-infected hepatocytes, and rapid hepatocyte proliferation during early life, potentially limiting viral integration events and promoting cccDNA clearance (18). Furthermore, prolonged HBsAg exposure in older children may negatively impact HBV-specific T cell populations, leading to diminished antiviral immune responses (19). Thus, the observed age-dependent treatment outcomes might reflect not only chronological age but also a longer duration of infection before treatment initiation.

Breakthrough event analysis revealed striking disparities: 3.6% in the HBsAg loss group vs. 31.4% in the non-HBsAg loss group. Similar age-dependent patterns emerged, with significantly lower breakthrough rates in the younger cohort (12.07% vs. 34.43%). While reduced breakthrough rates in the HBsAg loss group align with expectations given the association between viral suppression and treatment response, the markedly lower rates in younger patients suggest inherently enhanced therapeutic responsiveness. Furthermore, breakthrough events, occurring exclusively in lamivudine-treated patients (23.5%, *n* = 28), corroborate previous findings. Historical data indicates cumulative lamivudine resistance rates reaching 29% after 5 years of therapy (20). In contrast, entecavir demonstrates excellent long-term efficacy with minimal resistance (20, 21), while tenofovir exhibits exceptionally low resistance profiles (22, 23).

While sustained HBsAg loss represents the optimal therapeutic endpoint for NUCs therapy, achieving this outcome remains challenging. Current therapeutic guidelines recommend NUCs discontinuation following CR achievement and minimum 12-month consolidation therapy, with recent APASL guidelines extending this duration to 3 years (2, 3, 9). However, our findings suggest that children under 6 years demonstrating early HBeAg loss may benefit from extended NUCs therapy beyond these recommendations (7), aligning with EASL guidelines that acknowledge continued NUCs therapy until HBsAg clearance as a safe therapeutic endpoint (2).

HBV genotypes show distinct geographical distributions and clinical characteristics (24), with genotype C predominating in South Korea (>95% prevalence) (25, 26). In our cohort, patients experiencing breakthrough underwent concurrent YMDD (tyrosine-methionine-aspartate-aspartate) mutation testing and genotyping, uniformly demonstrating genotype C (7). While existing literature suggests superior treatment responses in genotypes A and B compared to genotypes D and C (27), our study demonstrated favorable treatment outcomes in young children. Recent Chinese investigations demonstrated favorable outcomes in younger children despite approximately two-thirds harboring genotype C (5). These findings suggest that the enhanced treatment responses observed in younger children likely transcend genotypic influences. It is also worth considering that pre-core or basal core promoter mutations, which can alter HBeAg dynamics independently of treatment response, occur with varying frequencies depending on the HBV genotype (28). For instance, genotype D has been reported to develop these mutations more frequently; thus, the prognostic value of HBeAg titer dynamics observed in our study might differ in patients with other genotypes.

This investigation acknowledges several limitations. First, the employed HBeAg assay provides semi-quantitative rather than fully quantitative results, potentially limiting analytical precision. Additionally, absolute values may vary between different manufacturers' reagents. Second, the evolution of HBV DNA testing methodologies during the study period resulted in varying upper detection limits, potentially impacting analysis. However, the fundamental importance of maintaining undetectable levels suggests minimal bias in CR assessment or historical data comparison. Third, temporal trends in initial antiviral therapy demonstrated age-related variations, with increased entecavir and tenofovir utilization in older age groups. The predominance of lamivudine likely reflects its extended history of pediatric approval and usage. These age-dependent prescription patterns, driven by drug approval timelines and guideline evolution, complicate direct inter-drug comparisons (29). Fourth, most patients enrolled in this study had viral genotype C; thus, our findings may not be applicable to children of different ethnic backgrounds with other viral genotypes. Fifth, quantitative HBsAg level testing was restricted due to Korean national health insurance policies, preventing its inclusion in this study, although prior studies have revealed its predictive value for HBsAg clearance.

Of particular clinical relevance, our model utilizes semi-quantitative HBeAg measurements that are readily available in routine clinical practice. While quantitative HBeAg assays exist, their high cost and limited availability restrict their practical application. In contrast, semi-quantitative HBeAg measurements are widely available, cost-effective, and already integrated into regualar clinical workflows. This practical advantage ensures that our predictive model can be immediately implemented in real-world clinical settings without requiring additional specialized testing. Future research should focus on validating our predictive model across larger, multicenter cohorts with diverse populations and various HBV genotypes.

## 5 Conclusions

This study suggests that early HBeAg titer dynamics may serve as a valuable predictor of HBsAg clearance in pediatric CHB patients. By incorporating both HBeAg titer reduction patterns and age at treatment initiation, we developed a practical predictive model for assessing HBsAg clearance probability. This novel approach, utilizing readily available semi-quantitative HBeAg measurements, could potentially assist clinicians in therapeutic decision-making and individualized treatment optimization in pediatric CHB patients, pending further validation. Additionally, our findings suggest that young children demonstrating early HBeAg loss might benefit from extended NUCs therapy beyond current recommendations.

## Data Availability

The raw data supporting the conclusions of this article will be made available by the authors, without undue reservation.
