# Supplementary material for: Predictive value of HBeAg titer dynamics for HBsAg clearance in pediatric chronic hepatitis B
Source: Front Pediatr. 2025 Apr 3;13:1539300. doi: 10.3389/fped.2025.1539300 (PMC12003423; doi:10.3389/fped.2025.1539300)
Supplement: Supplementary file 1 [file Datasheet1.pdf]

Supplementary Material

(Example Case 1)

Age at Start of Treatment: 3 years

Initial HBeAg Titer (1st titer): 1000 S/CO

HBeAg Titer at 6 months (2nd titer): 0.5 S/CO

*Calculation:

Calculate Log Reduction: $log\_reduction = \ln(1000+1) - \ln(0.5+1) = 6.908 - 0.405 = 6.503$

Apply Logistic Regression Model: $\text{Log Odds} = -1.182 + 0.308 \times 6.503 - 0.205 \times 3 = 0.204$

*Convert Log Odds to Probability:

$\text{Probability} = 1/(1+e^{-0.204}) \approx 0.551$

Predicted Probability of HBsAg Loss: 55.1%

*Interpretation:

By applying the optimal cutoff value of 0.195 (19.5%) derived from the ROC curve, this patient is considered to have a high likelihood of achieving HBsAg clearance.

(Example Case 2)

Age at Start of Treatment: 10 years

Initial HBeAg Titer (1st titer): 1500 S/CO

HBeAg Titer at 12 months (2nd titer): 500 S/CO

*Calculation:

Calculate Log Reduction: $log\_reduction = \ln(1500+1) - \ln(500+1) = 7.313 - 6.216 = 1.097$

Apply Logistic Regression Model: $\text{Log Odds} = -1.182 + 0.308 \times 1.097 - 0.205 \times 10 = -2.894$

*Convert Log Odds to Probability:

$\text{Probability} = 1/(1+e^{-2.894}) \approx 0.052$

Predicted Probability of HBsAg Loss: 5.2%

*Interpretation:

By applying the optimal cutoff value of 0.195 (19.5%) derived from the ROC curve, this patient is considered to have a low likelihood of achieving HBsAg clearance.

HBsAg Loss Probability Calculator

Use this calculator to estimate the probability of HBsAg loss in patients undergoing treatment for hepatitis

Age (years): [ ]

1st HBeAg Titer (S/CO): [ ]

2nd HBeAg Titer (S/CO): [ ]

➔ Calculate Probability

The logistic regression model used is:

Log Odds = -1.182 + 0.308 × log_reduction - 0.205 × age

Where:

- log_reduction = ln(1st titer + 1) - ln(2nd titer + 1)
- age = Age at treatment initiation
- 1st titer = Initial HBeAg titer value at the start of treatment
- 2nd titer = HBeAg titer value at 12 months or at the time of negativization within 12 months

Probability = 1 / (1 + e^(-Log Odds))

The optimal cutoff value derived from the ROC curve is 19.5%.
